# Reinforcement Learning for Secure Semantic LEO Satellite Networks: Joint Fidelity-Secrecy Power Allocation

**DOI:** 10.3390/s26082546

**Published:** 2026-04-21

**Authors:** Feifei Zhou, Xiaorong Zhu

**Affiliations:** 1 College of Telecommunications and Information Engineering, Nanjing University of Posts and Telecommunications, Nanjing 210003, China; xrzhu@njupt.edu.cn; 2 State Grid Electric Power Research Institute, Nari Group Co., Ltd., Nanjing 211106, China

**Keywords:** semantic communications, LEO satellite networks, physical-layer security, semantic fidelity, reinforcement learning

## Abstract

Semantic communications have emerged as a key paradigm for intelligent sixth-generation (6G) wireless networks, which aim to convey the meaning of information rather than accurate bit sequences. However, in open-space low Earth orbit (LEO) satellite links, the broadcast nature and wide beam coverage expose semantic transmissions to severe eavesdropping risks. This paper establishes a unified theoretical and algorithmic framework for secure semantic downlink transmission in satellite networks. In particular, we first develop an integrated mathematical model that couples the semantic representation process, physical-layer satellite propagation characteristics, and information-theoretic secrecy into a single analytical formulation. By defining a joint semantic security cost function, the antagonistic trade-off between semantic fidelity and secrecy capacity is quantitatively characterized under realistic power, beamforming, and propagation constraints. To balance semantic fidelity and information secrecy, a reinforcement-learning-based optimization framework is proposed, wherein an actor–critic agent learns optimal power allocation and semantic weighting strategies through continuous interaction with the environment. This learning-based optimization approach enables autonomous control without requiring explicit channel distribution knowledge or offline parameter tuning. Extended simulation results show that the proposed approach consistently enhances both semantic fidelity and secrecy performance compared with conventional power-control schemes and demonstrate its potential as a foundational architecture for secure and intelligent semantic communications in next-generation satellite networks.

## 1. Introduction

### 1.1. Background

The evolution toward sixth-generation (6G) wireless networks envisions a seamless integration of terrestrial and non-terrestrial infrastructures, thereby forming satellite–terrestrial integrated networks (STINs) that provide global, intelligent connectivity [1,2,3]. In such systems, satellites play a pivotal role in extending broadband coverage to remote and maritime regions, which can support emergency communications [4] and enable massive Internet-of-things (IoT) services [5,6,7]. However, the vast data volumes generated by heterogeneous sources—including sensory streams, multimedia content, and situational awareness information—create severe transmission burdens on bandwidth-limited satellite links [8,9,10]. Conventional communication systems designed under Shannon’s information theory primarily aim to minimize bit error rate (BER) or maximize spectral efficiency, regardless of the underlying meaning of the transmitted data [11,12,13]. This bit-level paradigm leads to massive redundancy, especially when the receiver’s task is semantic, e.g., classification, inference, or description [14].

Semantic communication has recently emerged as a transformative paradigm that redefines the objective of communication systems from bit-level accuracy to meaning-level effectiveness [15,16,17]. This paradigm originates from Weaver’s extension of Shannon theory and has been realized by advances in deep learning and natural language processing, where semantic encoders and decoders can learn compact latent representations that capture the essential content of raw messages [18,19,20]. By mapping high-dimensional data into task-relevant semantic embeddings, semantic communication systems can significantly reduce transmission redundancy, improve robustness against channel impairments, and enable goal-oriented communication, such as inference, classification, or decision making, directly at the receiver side [21,22,23]. Recent studies [24,25,26] have demonstrated that semantic encoding can achieve substantial gains in bandwidth efficiency and task accuracy, particularly under resource-constrained or noisy environments, thus marking it as a key enabler for intelligent 6G communication networks. Nevertheless, the open and broadcast nature of satellite downlink channels introduces inherent security vulnerabilities [27,28,29]. Due to the long-distance propagation and wide beam coverage, malicious eavesdroppers can easily intercept semantic signals, potentially inferring sensitive information even from partially decoded representations [30,31,32]. Therefore, future non-terrestrial networks must simultaneously achieve semantic efficiency and information secrecy to ensure that the extracted meaning remains accurate for legitimate users while being unintelligible to adversaries in an efficient manner. This dual requirement motivates the need for a unified semantic security framework that holistically models, analyzes, and optimizes the trade-off between fidelity and secrecy under realistic satellite constraints.

### 1.2. Related Works

LEO satellite networks are envisioned as key enablers of STINs for 6G systems, which provide global coverage, broadband backhaul, and ubiquitous connectivity for maritime, remote, and aerial users. Despite these advantages, LEO channels exhibit distinct characteristics that make efficient and secure communication challenging [2]. The stringent spectrum and power budgets onboard satellites limit data throughput, while orbital motion induces rapid channel variations and Doppler shifts, complicating channel estimation and synchronization [33]. In [34], the authors proposed a joint optimization framework for beam direction and multi-dimensional resource allocation in dynamic multi-beam LEO satellite networks to maximize long-term user sum rate with fairness. In [35], the authors proposed joint task offloading and resource allocation in satellite mobile edge computing systems with heterogeneous IoT task demands to improve user utility. Moreover, the extremely wide beam coverage of LEO downlinks creates large exposure regions, thus allowing unintended receivers or adversaries to intercept transmissions easily [36]. Traditional Shannon-based communication designs, which focus on minimizing symbol error rate or maximizing bit-level capacity, are inherently inefficient for such settings, since they ignore the fact that transmitted bits could be semantically redundant with respect to the users’ tasks [37]. To address these limitations, semantic communication has emerged as a promising paradigm that shifts the objective from reliable bit delivery to effective meaning transmission. Recent studies such as [15,38] have proposed end-to-end neural architectures that learn semantic encoders and decoders to capture task-relevant latent representations, and achieved significant gains in compression efficiency, robustness, and task accuracy compared with conventional systems. In [11], the authors propose a novel semantic quantification methods over bits for more efficient semantic transmission. These approaches [11,15,38] demonstrate that by transmitting only the semantically significant components of information, communication systems can significantly reduce bandwidth and energy consumption. However, most of these semantic communication works are limited to terrestrial environments, e.g., cellular or Wi-Fi networks [15], where channels are relatively stable and local interference dominates. When applied to LEO satellite downlinks characterized by strong line-of-sight (LoS) propagation and high mobility, semantic systems face additional challenges, including long propagation delays [39], beam misalignment [40], and increased vulnerability [41] to signal interception over large geographic areas.

The open and broadcast nature of satellite channels raises critical concerns regarding privacy protection and information confidentiality. In this context, a great amount of attention has been paid to satellite physical-layer security (PLS) [42], where techniques such as intelligent reflective surface (IRS) [43], artificial noise (AN) injection [44], cooperative jamming [45], and directional beamforming [46] have been developed to create signal-to-noise ratio (SNR) advantages for legitimate users over eavesdroppers. These methods [44,45,46] effectively suppress interception at the symbol level and improve secrecy capacity under Rician or LoS fading conditions. Nevertheless, conventional PLS frameworks operate strictly within the Shannon bit-pipe abstraction, optimizing secrecy rate metrics without considering the semantic meaning or task relevance of the transmitted information. In [47], the authors considered the semantic integrity the protection of semantic-level semantics and proposed a novel cross-layer semantic security metric. However, the intersection between semantic communication and satellite PLS introduces new and intricate research questions. Semantic representations often encode high-level contextual and relational knowledge. Thus, even partially decoded semantic features can leak sensitive information about the original content or task [48]. Conversely, excessive artificial noise or aggressive power randomization designed to improve secrecy may distort the latent semantic space and degrade task performance. This mutual interference between semantic fidelity and secrecy makes existing separate treatments of semantic efficiency and physical-layer confidentiality inadequate for practical satellite systems. Hence, a unified perspective is therefore required to understand and optimize the trade-off between semantic distortion and secrecy risk.

Several recent works have started to bridge semantic communications with emerging satellite/NTN architectures and resource allocation paradigms. The authors in [40] investigate a SAGIN setting and propose a DRL-based resource allocation framework for hybrid bit-level and generative semantic image delivery. It highlights the role of learning-based policies in dynamic multi-layer space–air–ground environments. The authors in [49] study semantic communication in a satellite-borne edge cloud network for computation offloading, where semantic coding and learning-based updating mechanisms are leveraged to reduce latency/energy under resource constraints. From a system architecture perspective, the authors in [50] discuss self-organized network principles for 5G mega-constellations, providing a forward-looking view on how large-scale LEO systems may support advanced service orchestration. Moreover, computation offloading in multi-layer/multi-constellation LEO environments [51] has been considered, where hierarchical learning and network selection are used to optimize end-to-end latency and reliability. Compared with these works, our paper focuses on physical-layer security for semantic communication and develops an actor–critic RL approach to jointly optimize transmit power and semantic power allocation under a wiretap model, explicitly characterizing the semantic fidelity–secrecy capacity tradeoff.

### 1.3. Contributions

Motivated by the above-mentioned challenges, this work develops an integrated framework that couples semantic representation, satellite propagation modeling, and AN-aided secure transmission within a joint optimization structure. The proposed formulation explicitly quantifies how semantic fidelity and secrecy capacity depend on physical control variables, namely transmit power and semantic-to-noise power ratio, and further introduces a reinforcement learning (RL)-based optimizer that adapts these parameters online under dynamic LEO channel conditions. In doing so, this study bridges the gap between semantic efficiency and physical-layer security, thus offering a new direction for secure and intelligent semantic communications in next-generation satellite networks. The main contributions of this paper are summarized as follows:

- We establish a mathematical model that integrates semantic representation, satellite propagation characteristics, and artificial-noise-aided transmission, which provides a foundation for jointly analyzing semantic and security performance. A novel cost function is formulated to couple semantic distortion, secrecy leakage, and power efficiency, thereby enabling quantitative evaluation of the fidelity–secrecy trade-off under realistic energy and beamforming constraints.
- Based on the derived SINR expressions, the optimal semantic power allocation and total transmit power are analytically characterized, revealing the structural dependence between semantic accuracy and secrecy robustness in LEO channels.
- We develop an actor–critic-based RL approach to autonomously learn near-optimal transmission strategies through online interaction for a dynamic balance between semantic fidelity and physical-layer security.
- Extended simulation results show that the proposed approach consistently enhances both semantic fidelity and secrecy performance compared with conventional power-control schemes.

## 2. Satellite Downlink Channel and Transmission Model

**Figure 1:**
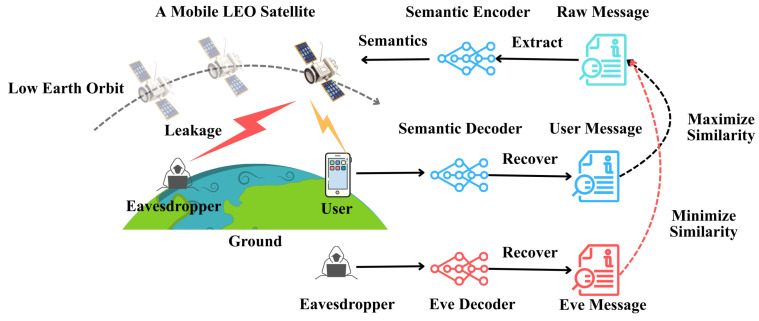
The considered semantic LEO satellite communication system with the presence of eavesdropping.

We consider a low Earth orbit (LEO) satellite *S* equipped with an $N_{t}$-element phased array, transmitting downlink signals to a legitimate ground terminal *U* in the presence of a passive eavesdropper *E*, as shown in Figure 1. The system operates in the Ka-band, where propagation is dominated by a near line-of-sight (LoS) component and weak tropospheric scattering. The downlink configuration is adopted since the satellite possesses higher transmission power, centralized control, and beamforming capability, whereas the ground terminals have limited energy and primarily perform semantic reconstruction. This asymmetry also highlights the need for effective AN injection to enhance physical-layer secrecy.

### 2.1. Propagation Environment and Channel Model

The propagation path between the satellite and a ground node i∈{U,E} is modeled as a composite of large-scale attenuation, small-scale Rician fading, and Doppler-induced time variation due to the relative motion of the satellite. Under the assumption of accurate carrier and timing synchronization at the receiver, the complex baseband equivalent channel can be expressed as

(1) $$h_{i}\left(t\right)=\sqrt{\beta_{i}\left(t\right)}\;g_{i}\left(t\right)\;e^{-j2\pi (f_{c}\tau_{i}\left(t\right)+f_{D,i}t)},$$

where $f_{c}$ is the carrier frequency, $\tau_{i}\left(t\right)=d_{i}\left(t\right)/c$ is the propagation delay with *c* denoting the speed of light, $\beta_{i}\left(t\right)$ captures large-scale attenuation, $g_{i}\left(t\right)$ represents the small-scale fading component, and $f_{D,i}$ is the instantaneous Doppler frequency shift. The Doppler shift arises from the relative velocity between the satellite and the ground terminal, given by

(2) $$f_{D,i}=\frac{v_{\mathrm{rel},i}}{c}\;f_{c}\;\cos\varphi_{i},$$

where $v_{\mathrm{rel},i}$ is the relative speed along the line-of-sight (LoS) direction, and $\varphi_{i}$ is the angle between the satellite’s velocity vector and the propagation direction.

The slant range between the satellite and node *i* is determined by spherical geometry as

(3) $$d_{i}\left(t\right)=\sqrt{R_{E}^{2}+{\left(R_{E}+H\right)}^{2}-2R_{E}\left(R_{E}+H\right)\cos\theta_{i}\left(t\right)},$$

where $R_{E}$ denotes the Earth’s radius, *H* the satellite altitude, and $\theta_{i}\left(t\right)$ the Earth central angle corresponding to the time-varying ground elevation. The rate of change of $d_{i}\left(t\right)$ determines $v_{\mathrm{rel},i}$ in (2), linking geometric dynamics with frequency offset.

The large-scale gain $\beta_{i}\left(t\right)$ is expressed as

(4) $$\beta_{i}\left(t\right)=\frac{G_{t}G_{r,i}}{L_{\mathrm{fs}}\left(d_{i}\left(t\right),f_{c}\right)\;L_{\mathrm{atm}}\left(\theta_{i}\left(t\right),f_{c}\right)\;L_{\mathrm{rain}}\left(\theta_{i}\left(t\right),f_{c}\right)},$$

where $G_{t}$ and $G_{r,i}$ are the satellite transmit and ground receive antenna gains in linear scale. The free-space loss is $L_{\mathrm{fs}}\left(d_{i},f_{c}\right)={\left(4\pi d_{i}f_{c}/c\right)}^{2}$, while $L_{\mathrm{atm}}$ and $L_{\mathrm{rain}}$ denote atmospheric absorption and rain attenuation, respectively, modeled according to ITU-R P.676 and P.838. These terms vary slowly compared with the symbol duration and are incorporated into $\beta_{i}\left(t\right)$ as quasi-static components.

The small-scale fading component $g_{i}\left(t\right)$ follows a Rician distribution:

(5) $$g_{i}\left(t\right)=\sqrt{\frac{K_{i}}{K_{i}+1}}\;g_{i}^{\left(\mathrm{LoS}\right)}\left(t\right)+\sqrt{\frac{1}{K_{i}+1}}\;g_{i}^{\left(\mathrm{NLoS}\right)}\left(t\right),$$

where $K_{i}$ is the Rician factor representing the power ratio between LoS and scattered components. The deterministic term $g_{i}^{\left(\mathrm{LoS}\right)}\left(t\right)$ corresponds to the array steering response aligned with the time-varying satellite position, while the scattered component $g_{i}^{\left(\mathrm{NLoS}\right)}\left(t\right)∼\mathrm{CN}\left(0,1\right)$ models diffuse multipath effects. In LEO downlink channels, $K_{i}\;\gg \;1$, meaning the LoS component dominates and small-scale variations mainly arise from Doppler dynamics rather than rich multipath scattering.

### 2.2. Downlink Signal Mapping and Power Allocation

Given the time-varying channel model with Doppler effects, the satellite transmits both semantic information and AN in each symbol interval to jointly ensure meaning fidelity and physical-layer confidentiality. Let the semantic baseband symbol be denoted as u∈C with normalized power $E[|u{|}^{2}]=1$. The $N_{t}$-element satellite array forms the transmitted vector as

(6) $$x\left(t\right)=\sqrt{\rho P_{t}}\;w_{s}\;u\left(t\right)+\sqrt{\left(1-\rho \right)P_{t}}\;w_{n}\;n_{\mathrm{AN}}\left(t\right),\;0\leq \rho \leq 1,$$

where $P_{t}$ denotes the instantaneous transmit power; ρ is the semantic power allocation ratio; $w_{s},w_{n}\in C^{N_{t}}$ are unit-norm and mutually orthogonal beamforming vectors satisfying $w_{s}^{H}w_{n}=0$; and $n_{\mathrm{AN}}\left(t\right)\;∼\;\mathrm{CN}\left(0,1\right)$ denotes the artificial noise sequence. The orthogonality constraint ensures that the AN is radiated in a spatial null space with respect to the main semantic beam, thereby minimizing interference to the legitimate user while degrading the eavesdropper’s effective channel.

The received baseband signals at the legitimate user *U* and the eavesdropper *E* can thus be expressed as

(7) $$y_{i}\left(t\right)=h_{i}^{H}\left(t\right)\;x\left(t\right)+n_{i}\left(t\right),\;i\in \left\{U,E\right\},$$

where $h_{i}\left(t\right)$ follows the time-varying model in (1) that includes Doppler phase rotation, and $n_{i}\left(t\right)\;∼\;\mathrm{CN}\left(0,N_{0}\right)$ represents additive white Gaussian noise. Then, substituting (6) into (7), we have

(8) $$\begin{array}{cc} y_{U}\left(t\right) & =\sqrt{\rho P_{t}}\;h_{U}^{H}\left(t\right)w_{s}\;u\left(t\right)+\sqrt{\left(1-\rho \right)P_{t}}\;h_{U}^{H}\left(t\right)w_{n}\;n_{\mathrm{AN}}\left(t\right)+n_{U}\left(t\right), \end{array}$$

(9) $$\begin{array}{cc} y_{E}\left(t\right) & =\sqrt{\rho P_{t}}\;h_{E}^{H}\left(t\right)w_{s}\;u\left(t\right)+\sqrt{\left(1-\rho \right)P_{t}}\;h_{E}^{H}\left(t\right)w_{n}\;n_{\mathrm{AN}}\left(t\right)+n_{E}\left(t\right). \end{array}$$

By defining the beamformed power gains ${\hat{\Gamma }}_{i}\left(t\right)={\left|h_{i}^{H}\left(t\right)w_{s}\right|}^{2}$ and ${\hat{\Delta }}_{i}\left(t\right)={\left|h_{i}^{H}\left(t\right)w_{n}\right|}^{2}$, the instantaneous signal-to-interference-plus-noise ratio (SINR) at node *i* can be represented by

(10) $$\gamma_{i}\left(t\right)=\frac{\rho P_{t}{\hat{\Gamma }}_{i}\left(t\right)}{\left(1-\rho \right)P_{t}{\hat{\Delta }}_{i}\left(t\right)+N_{0}}.$$

The SINR formulation (10) inherently reflects the joint impact of transmit power control, semantic-to-noise power allocation, and Doppler-induced channel fluctuation. An increase in ρ enhances the semantic signal strength received by the legitimate user, thereby improving semantic reconstruction accuracy, whereas reducing ρ allocates more power to AN, effectively lowering the eavesdropper’s SINR and improving confidentiality. In LEO scenarios, Doppler variations cause rapid phase rotation in $h_{i}\left(t\right)$, which leads to temporal SINR fluctuations even for fixed ρ and $P_{t}$. Therefore, adaptive power allocation strategies are essential to dynamically balance semantic fidelity and secrecy capacity under such non-stationary satellite channels.

## 3. Semantic Representation and Transmission Model

### 3.1. Semantic Encoder

Let $M\in R^{n}$ denote the original message, e.g., a telemetry segment or textual token, sampled from a source with density $p_{M}\left(\cdot \right)$ and finite differential entropy H(M). The satellite implements a low-complexity semantic encoder $f_{\theta }:R^{n}\;\to \;R^{k}$ that extracts a *k*-dimensional latent vector

(11) $$s\;=\;f_{\theta }\left(M\right)\;=\;W_{2}\;\sigma \left(W_{1}M+b_{1}\right)+b_{2},$$

where $W_{1}\;\in \;R^{h\times n}$, $W_{2}\;\in \;R^{k\times h}$, $b_{1}\;\in \;R^{h}$, $b_{2}\;\in \;R^{k}$, and σ(·) is ReLU to capture component-wise nonlinearity. The parameter set is $\theta =\{W_{1},W_{2},b_{1},b_{2}\}$. To capture multi-topic semantics while enabling tractable second-order analysis, we assume the latent distribution follows a Gaussian-mixture prior

(12) $$p\left(s\right)\;=\;\sum_{\ell =1}^{L}\pi_{\ell }\;N\left(s\;|\;\mu_{\ell },\Sigma_{\ell }\right),\;\pi_{\ell }\geq 0,\;\sum_{\ell }\pi_{\ell }=1,$$

and define the latent covariance

(13) $$\Sigma_{s}≜E\left[\left(s-Es\right){\left(s-Es\right)}^{\;⊤}\right].$$

To decouple semantic statistics from the physical-layer energy budget, the encoder applies a normalization layer so that

(14) $$\tilde{s}\;=\;\Sigma_{s}^{-1/2}\left(s-Es\right),\;E\left[\tilde{s}\right]=0,\;\;E{∥\tilde{s}∥}^{2}=k,$$

followed by a per-block power shaping that enforces the semantic feature power constraint:

(15) $$E\left[∥\tilde{s}{∥}^{2}\right]\;=\;k\;⟹\;E\left[{∥u∥}^{2}\right]\;=\;1,\;u≜q^{⊤}\tilde{s},$$

where $q\;\in \;R^{k}$ is a unit-norm projection that maps the *k*-dimensional latent to a scalar complex symbol stream, defined per channel use, to be transmitted over one spatial beam. Consequently, the encoder optimization used offline prior to deployment is represented by

(16) $$\min_{\theta ,q}\;\;E\;\left[∥M-\hat{M}{∥}^{2}\right]{\;s.t.\;E[|u|}^{2}]=1,$$

where $\hat{M}$ denotes the end-to-end reconstruction at the receiver, as defined in (17). Problem (16) fixes the latent power at baseband and leaves the physical-layer energy allocation to the downlink mapper via $\left(\rho ,P_{t}\right)$.

### 3.2. Semantic Decoder

The ground user applies a lightweight semantic decoder $g_{\phi }:C\;\to \;R^{n}$ to reconstruct the meaning from the received symbols $y_{U}$. The structurally symmetric decoder can be represented by

(17) $$\hat{M}\;=\;g_{\phi }\left(y_{U}\right)\;=\;V_{2}\;\sigma \;\left(V_{1}\;y_{U}+c_{1}\right)+c_{2},$$

with $\phi =\{V_{1},V_{2},c_{1},c_{2}\}$ and the same nonlinearity σ(·) as defined in (11). The end-to-end semantic flow obeys the Markov chain

(18) $$M\;\overset{\;f_{\theta }\;}{\to }\;s\;\overset{\;q^{⊤}\;}{\to }\;u\;\overset{\;\mathrm{downlink}\;}{\to }\;y_{U}\;\overset{\;g_{\phi }\;}{\to }\;\hat{M},$$

which implies $I\left(M;\hat{M}\right)\leq I\left(M;u\right)\leq I\left(u;y_{U}\right)$ by data processing. Since $E[|u{|}^{2}]=1$ by (15), the mutual information $I(u;y_{U})$ is determined solely by the SINR $\gamma_{U}$ at the physical layer.

### 3.3. Semantic Fidelity and Secrecy

To connect the meaning-level fidelity to the physical-layer reliability, we use a widely adopted monotone approximation that maps SINR to semantic distortion:

(19) $$D_{\mathrm{sem}}\left(\gamma_{U}\right)\approx \frac{\kappa_{\mathrm{SF}}}{\log_{2}\left(1+\gamma_{U}\right)},\;\kappa_{\mathrm{SF}}>0,$$

which yields $SF\left(\gamma_{U}\right)\approx 1-\kappa_{\mathrm{SF}}/\log_{2}\left(1+\gamma_{U}\right)$.

We quantify semantic fidelity as the normalized mutual information between the source meaning and its reconstruction, represented by

(20) $$\mathrm{SF}\;≜\;\frac{I(M;\hat{M})}{I(M;M)}\;=\;1-\frac{H(M|\hat{M})}{H(M)}\;\in \;\left[0,1\right],$$

where I(·;·) and H(·) denote mutual and differential entropy, respectively. Under small-distortion operation and smooth decoders, a second-order approximation together with the information-minimum mean square error (I-MMSE) relation builds a monotone link between fidelity and the effective SNR, represented by

(21) $$\mathrm{SF}\left(\gamma_{U}\right)\;\approx \;1-\frac{\kappa }{\log_{2}\left(1+\gamma_{U}\right)},\;\kappa >0,$$

where κ aggregates the semantic compression depth via k/n, task difficulty through the curvature of $g_{\phi }$, and the source entropy scale H(M). The approximation (21) is used to connect meaning-level distortion to physical-layer reliability while preserving the correct low- and high-SNR asymptotes.

To characterize confidentiality, we adopt the instantaneous secrecy capacity of the wiretap link driven by the physical SINRs:

(22) $$C_{s}\;=\;{\left[\log_{2}\left(1+\gamma_{U}\right)-\log_{2}\left(1+\gamma_{E}\right)\right]}^{+},$$

which measures the maximum secure rate in the unit of bits/s/Hz, at which the semantic stream can be conveyed without information leakage, given $\left(\rho ,P_{t},w_{s},w_{n}\right)$ and the channel realization $\left(h_{U},h_{E}\right)$. Both fidelity and secrecy are functions of the control variables:

(23) $$\mathrm{SF}\;=\;\mathrm{SF}\left(\rho ,P_{t};{\hat{\Gamma }}_{U},{\hat{\Delta }}_{U}\right),\;C_{s}\;=\;C_{s}\left(\rho ,P_{t};{\hat{\Gamma }}_{U},{\hat{\Delta }}_{U},{\hat{\Gamma }}_{E},{\hat{\Delta }}_{E}\right),$$

thereby establishing the semantic–security coupling used later in the joint objective. In particular, increasing ρ improves $\gamma_{U}$ but can also increase $\gamma_{E}$, thus reducing $C_{s}$ in (22). Conversely, decreasing ρ allocates more power to AN, typically enlarging $C_{s}$ while degrading SF. This antagonistic dependence is the fundamental trade-off that motivates the co-design developed in the optimization section.

## 4. Joint Semantic-Security Optimization

The preceding sections have demonstrated that both semantic fidelity and secrecy capacity depend critically on two coupled control parameters: the total transmit power $P_{t}$ and the semantic power allocation ratio ρ. Increasing ρ enhances the semantic SNR at the legitimate user and thus improves the meaning reconstruction accuracy, but it also increases the eavesdropper’s SNR and weakens secrecy. Conversely, reducing ρ devotes more energy to AN, thereby suppressing the eavesdropper at the cost of higher semantic distortion. Meanwhile, the total transmit power $P_{t}$ jointly determines the overall system energy efficiency, further complicating the trade-off among semantic fidelity, secrecy performance, and power consumption. This section formulates a unified optimization model to characterize these competing objectives.

### 4.1. A. Composite Cost Function Design

To quantify the semantic–security trade-off, we introduce a composite objective function that integrates semantic distortion, secrecy risk, and energy consumption by:

(24) $$J\left(\rho ,P_{t}\right)=\underset{\mathrm{semantic}\;\mathrm{distortion}}{\underset{︸}{\frac{\kappa }{\log_{2}\left(1+\gamma_{U}\right)}}}+\underset{\mathrm{secrecy}\;\mathrm{penalty}}{\underset{︸}{\mu e^{-\rho P_{t}{\left|h_{E}\right|}^{2}}}}+\underset{\mathrm{power}\;\mathrm{cost}}{\underset{︸}{\lambda \frac{P_{t}}{P_{\max}}}},$$

where κ, μ, and λ are positive weighting coefficients that determine the relative importance of semantic fidelity, secrecy enhancement, and power efficiency, respectively. The first term represents the inverse of semantic fidelity, $\mathrm{SF}\left(\gamma_{U}\right)=1-\kappa /\log_{2}\left(1+\gamma_{U}\right)$, which penalizes low reconstruction quality at the legitimate receiver. A larger $\gamma_{U}$, achieved via increased ρ or $P_{t}$, reduces this term and thus improves semantic performance. The second term, $\mu e^{-\rho P_{t}{\left|h_{E}\right|}^{2}}$, characterizes the residual information leakage to the eavesdropper. Its exponential form stems from secrecy-outage analysis, reflecting the fact that the eavesdropper’s decoding probability decreases exponentially with increasing interference or AN power. The last term, $\lambda P_{t}/P_{\max}$, imposes a linear energy cost relative to the maximum available satellite transmit power $P_{\max}$, ensuring compliance with onboard power constraints and promoting energy-aware communication.

### 4.2. Optimization Problem Formulation

The joint semantic–security optimization problem can be expressed as

(25) $$\begin{array}{c} \min_{0\leq \rho \leq 1,\;0\leq P_{t}\leq P_{\max}}J\left(\rho ,P_{t}\right), \end{array}$$

where the decision variables ρ and $P_{t}$ jointly determine the effective SINRs of both the legitimate and eavesdropping channels:

(26) $$\gamma_{U}=\frac{\rho P_{t}{\left|h_{U}\right|}^{2}}{\left(1-\rho \right)P_{t}{\left|h_{U}^{H}w_{n}\right|}^{2}+N_{0}},\;\gamma_{E}=\frac{\rho P_{t}{\left|h_{E}\right|}^{2}}{\left(1-\rho \right)P_{t}{\left|h_{E}^{H}w_{n}\right|}^{2}+N_{0}}.$$

This formulation reflects a nonconvex coupled problem, where increasing semantic fidelity influences secrecy efficiency. The trade-off surface defined by $\left(\rho ,P_{t}\right)$ is highly nonlinear and channel-dependent, particularly under the time-varying propagation conditions of LEO satellite links.

The weighting factors (κ,μ,λ) provide system-level flexibility: for mission-critical tasks that demand high interpretability, e.g., remote-sensing data transmission, a larger κ emphasizes semantic accuracy. For secure communications, a higher μ favors secrecy, while for energy-limited satellites, an increased λ enforces power conservation. By tuning these parameters, the model can represent different operational modes, from semantics-prioritized transmission to secrecy-prioritized secure broadcasting.

The cost function $J(\rho ,P_{t})$ in (24) exhibits several important properties that make direct analytical optimization difficult yet suitable for learning-based control:

1. Nonconvexity and Coupling: The semantic term $\frac{\kappa }{\log_{2}\left(1+\gamma_{U}\right)}$ and the secrecy term $\mu e^{-\rho P_{t}{\left|h_{E}\right|}^{2}}$ are both nonlinear and jointly dependent on ρ and $P_{t}$, producing a nonconvex landscape with potentially multiple local minima.
2. Channel Uncertainty: The instantaneous channel coefficients $\left(h_{U},h_{E}\right)$ vary rapidly in LEO downlinks due to orbital motion and beam steering. Accurate real-time estimation of these parameters is costly and sometimes infeasible.
3. Dynamic Trade-off: The optimal power allocation $\left(\rho^{★},P_{t}^{★}\right)$ depends not only on channel states but also on mission objectives and system constraints that may evolve over time. This calls for an adaptive optimization mechanism rather than a static closed-form solution.

Considering these challenges, conventional convex optimization or analytical derivation cannot guarantee robust or real-time solutions in practical satellite environments. To address this issue, in the next section, we will develop an RL-based adaptive optimization framework, where the satellite acts as an intelligent agent that continuously observes the environment state, including semantic feedback and channel indicators, and learns the optimal power allocation policy $\left(\rho ,P_{t}\right)$ through interaction. This approach allows the system to approximate near-optimal behavior under uncertainty and dynamic conditions without explicit channel models or prior statistical knowledge.

## 5. Proposed Reinforcement-Learning-Based Online Optimization

To address the inherent nonconvexity and time-varying nature of the joint semantic-security optimization problem formulated in Section 5, we adopt an actor–critic-based RL approach that enables the satellite to autonomously adjust the transmit power $P_{t}$ and semantic ratio ρ in real time. The RL agent continuously observes the environment, evaluates transmission outcomes, and improves its policy through repeated interaction without requiring explicit knowledge of the underlying channel distributions. This data-driven adaptation is particularly suitable for nonstationary LEO satellite channels, where analytical solutions are impractical due to rapid variations in geometry, path loss, and fading.

### 5.1. MDP Formulation

The system environment is modeled as a Markov decision process (MDP) M=〈S,A,r,P〉, where S denotes the state space, A the action space, *r* the reward function, and P the state transition probability. At each time step *t*, the agent observes the environment state $s_{t}\in S$, executes an action $a_{t}\in A$, and receives an immediate reward $r_{t}$. The goal of the RL agent is to learn a stationary policy π(a|s) that maximizes the expected long-term discounted reward:

(27) $$J_{\mathrm{RL}}=E_{\pi }\;\left[\sum_{t=0}^{\infty }\gamma^{t}r_{t}\right],$$

where 0<γ<1 is the discount factor reflecting the temporal importance of future rewards. The MDP abstraction allows the satellite to treat dynamic semantic-security optimization as a sequential decision problem, capturing both temporal correlations, e.g., channel evolution, and performance feedback.

### 5.2. State Space

The state vector $s_{t}$ aggregates real-time physical, semantic, and security-related indicators observable by the satellite, defined as

(28) $$s_{t}=\left[{\hat{\Gamma }}_{U},\;{\hat{\Gamma }}_{E},\;f_{D},\;{\bar{C}}_{s}\left(t-1\right),\;\rho_{t-1},\;P_{t-1},\;\xi \left(t\right)\right].$$

Here, ${\hat{\Gamma }}_{U}$ and ${\hat{\Gamma }}_{E}$ represent the instantaneous beamformed channel gains of the legitimate user and the eavesdropper, respectively, which can be estimated through pilot feedback or side information from the network. The averaged secrecy capacity ${\bar{C}}_{s}\left(t-1\right)$ encapsulates the system’s long-term confidentiality level as observed in the previous time slot, providing historical feedback for stability. The pair $\left(\rho_{t-1},\;P_{t-1}\right)$ represents the previously applied semantic ratio and transmit power, allowing the agent to account for temporal correlations and avoid abrupt control actions. Finally, ξ(t) reflects the instantaneous semantic task weight, quantifying the contextual importance of the transmitted information, such as prioritizing critical command data over routine telemetry. Together, these elements ensure that the RL agent jointly perceives the physical-layer environment, semantic relevance, and security conditions, thereby enabling context-aware and adaptive power control decisions in dynamic satellite communication scenarios.

### 5.3. Action Space

The action taken by the agent at each step consists of adjusting both the semantic ratio and the transmit power:

(29) $$a_{t}=\left[\rho_{t},P_{t}\right]\in \left[0,1\right]\times \left[0,P_{\max}\right],$$

where $\rho_{t}$ controls the fraction of transmit power devoted to the semantic signal, and $P_{t}$ specifies the total transmission power for the current time slot. This compact continuous action space allows fine-grained adaptation of power allocation, consistent with hardware feasibility onboard satellites equipped with digital beamforming and power-amplifier control circuits.

### 5.4. Reward Function

The reward function is carefully designed to reflect the instantaneous system utility derived from semantic fidelity, secrecy preservation, and energy efficiency, as well as to enforce operational constraints. The reward received at time *t* is defined as

(30) $$\begin{array}{cc} r_{t}= & -\left[\frac{\kappa \;\xi (t)}{\log_{2}\left(1+\gamma_{U}\left(t\right)\right)}+\mu e^{-\rho_{t}P_{t}{\hat{\Gamma }}_{E}\left(t\right)}+\lambda \frac{P_{t}}{P_{\max}}\right]\\ \; & -\alpha {\left[C_{\min}-C_{s}\left(t\right)\right]}_{+}-\beta \left({\left[\rho_{t}-1\right]}_{+}+{\left[-\rho_{t}\right]}_{+}\right)-\beta^{\prime }\left({\left[P_{t}-P_{\max}\right]}_{+}+{\left[-P_{t}\right]}_{+}\right), \end{array}$$

where ${\left[x\right]}_{+}≜\max\left(x,0\right)$ denotes the ReLU operator. The first bracketed term penalizes the same cost components as in the offline objective $J(\rho ,P_{t})$ in (24), scaled by the semantic urgency factor ξ(t). The remaining terms impose soft penalties on constraint violations: (1) $\alpha {\left[C_{\min}-C_{s}\left(t\right)\right]}_{+}$ ensures that the instantaneous secrecy capacity $C_{s}\left(t\right)$ stays above the minimum acceptable threshold $C_{\min}$; and (2) β and $\beta^{\prime }$ penalize infeasible actions that exceed the allowable ranges of $\rho_{t}$ and $P_{t}$, ensuring stable exploration during training. This reward formulation ensures that maximizing expected cumulative reward is equivalent to minimizing long-term average cost while respecting physical and security constraints.

### 5.5. Learning Architecture

To solve the above continuous-state, continuous-action MDP, we adopt an actor–critic reinforcement learning framework with a Gaussian policy parameterization. The actor network parameterized by ϑ generates the mean $\mu_{\vartheta }\left(s\right)$ and covariance $\Sigma_{\vartheta }\left(s\right)$ of the Gaussian distribution:

(31) $$\begin{array}{cc} \pi_{\vartheta }\left(a|s\right) & =N\;\left(\mu_{\vartheta }\left(s\right),\;\Sigma_{\vartheta }\left(s\right)\right), \end{array}$$

(32) $$\begin{array}{cc} \nabla_{\vartheta }J_{\mathrm{RL}} & =E\;\left[\nabla_{\vartheta }\log\pi_{\vartheta }\left(a_{t}|s_{t}\right)\;{\hat{A}}_{t}+\eta_{H}\nabla_{\vartheta }H\left(\pi_{\vartheta }\right)\right], \end{array}$$

where ${\hat{A}}_{t}$ is the advantage function estimated by the critic, $\eta_{H}$ is the entropy regularization coefficient encouraging policy exploration, and $H(\pi_{\vartheta })$ denotes the entropy of the policy. The critic network parameterized by ψ estimates the value function $V_{\psi }\left(s_{t}\right)$, which is updated by minimizing the temporal difference (TD) loss

(33) $$L_{V}\left(\psi \right)=E\;\left[{\left(r_{t}+\gamma V_{\psi }\left(s_{t+1}\right)-V_{\psi }\left(s_{t}\right)\right)}^{2}\right].$$

This actor–critic architecture allows stable policy gradient updates, low variance in training, and online adaptability under the dynamic conditions of satellite networks.

### 5.6. Training Procedure

The training process proceeds in episodes, where each episode corresponds to one operational period, e.g., a satellite orbital window or a fixed number of time steps. During training, the agent continuously interacts with the environment, observes state transitions, collects experience tuples $\left(s_{t},a_{t},r_{t},s_{t+1}\right)$, and updates both actor and critic parameters using sampled trajectories. The training goal is to maximize the long-term expected cumulative reward:

(34) $$J_{\mathrm{RL}}=E_{\pi_{\vartheta }}\;\left[\sum_{t=0}^{\infty }\gamma^{t}r_{t}\right],$$

subject to the action and secrecy constraints described earlier.

The critic network first computes the TD target $y_{t}=r_{t}+\gamma V_{\psi }\left(s_{t+1}\right)$ and minimizes the loss $L_{V}\left(\psi \right)$ in (33). The advantage estimate is then obtained as ${\hat{A}}_{t}=y_{t}-V_{\psi }\left(s_{t}\right)$. The actor parameters are updated using the gradient ascent rule in (32), where the entropy term $\eta_{H}\nabla_{\vartheta }H\left(\pi_{\vartheta }\right)$ ensures continuous exploration during online adaptation. All network parameters are optimized using stochastic gradient descent or Adam with separate learning rates for the actor and critic.

This training process, as shown in Algorithm 1, allows the agent to iteratively learn a mapping from observed environment states to optimal power-control actions that minimize the long-term cost in (24). Since the learning is performed through continuous interaction with the physical communication environment, the resulting policy adapts naturally to temporal channel variations, semantic task shifts, and security-level requirements, enabling autonomous online optimization in real-world LEO satellite scenarios.
**Algorithm 1** RL-Based Online Semantic-Security Optimization  1: **Initialize:** actor parameters ϑ, critic parameters ψ.  2: **for** each episode e=1,…,E **do**  3:     Observe initial state $s_{0}$  4:     **for** each time step t=0,…,T **do**  5:          Sample action $a_{t}=\left[\rho_{t},P_{t}\right]∼\pi_{\vartheta }\left(a_{t}|s_{t}\right)$  6:          Project: $\rho_{t}\;\leftarrow \;\min\left\{1,\max\left\{0,\rho_{t}\right\}\right\}$, $P_{t}\;\leftarrow \;\min\left\{P_{\max},\max\left\{0,P_{t}\right\}\right\}$  7:          Execute $a_{t}$, observe reward $r_{t}$ and next state $s_{t+1}$  8:          Compute TD target: $y_{t}=r_{t}+\gamma V_{\psi }\left(s_{t+1}\right)$  9:          Compute advantage: ${\hat{A}}_{t}=y_{t}-V_{\psi }\left(s_{t}\right)$10:         Update critic: $\psi \;\leftarrow \;\psi -\eta_{c}\nabla_{\psi }L_{V}\left(\psi \right)$11:         Update actor: $\vartheta \;\leftarrow \;\vartheta +\eta_{a}\nabla_{\vartheta }\left[\log\pi_{\vartheta }\left(a_{t}|s_{t}\right){\hat{A}}_{t}+\eta_{H}H\left(\pi_{\vartheta }\right)\right]$12:     **end for**13: **end for**14: **Output:** trained policy $\pi_{\vartheta }\left(a|s\right)$ for online operation


## 6. Simulation Results

To validate the effectiveness of the proposed semantic security design and RL optimizer, numerical experiments are conducted in a semantic LEO satellite communication network. In particular, we consider a single-beam LEO satellite equipped with an $N_{t}=8$-element uniform linear array operating in the Ka band with carrier frequency $f_{c}=20\;\mathrm{GHz}$. The satellite altitude is H=600km, and the Earth radius is $R_{E}=6371\;\mathrm{km}$ [41,52]. The legitimate user and the eavesdropper are placed at elevation angles $\theta_{U}=60^{\circ }$ and $\theta_{E}=30^{\circ }$, respectively. The receiver noise power spectral density is $N_{0}=-174\;\mathrm{dBm}/\mathrm{Hz}$ with bandwidth B=10MHz. The maximum transmit power is $P_{\max}=40\;\mathrm{dBm}$, and the Rician factors are set to $K_{U}=12\;\mathrm{dB}$ and $K_{E}=6\;\mathrm{dB}$, representing dominant LoS and partially blocked conditions, respectively. The relative satellite velocity is $v_{\mathrm{rel}}=7.5\;\mathrm{km}/s$, resulting in a maximum Doppler shift $f_{D,\max}=500\;\mathrm{kHz}$ at $f_{c}=20\;\mathrm{GHz}$, which follows a cosine variation with elevation angle [53]. Atmospheric and rain attenuation are modeled by using ITU-R P.676 and P.838, giving a mean additional loss of 3–4dB for moderate rainfall rates of 25mm/h [54]. For the semantic module, the source messages are represented by n=128-dimensional feature vectors, compressed into k=32 dimensional latent vectors [47]. The semantic fidelity coefficient is set to κ=0.4, reflecting moderate semantic compression depth. In the joint cost function, the secrecy and energy weights are μ=0.8 and λ=0.05, respectively. The minimum required secrecy capacity threshold is $C_{\min}=1\;\mathrm{bit}/s/\mathrm{Hz}$. The RL agent implements an actor–critic architecture with fully connected neural networks of two hidden layers with sizes [256,128] and ReLU activation. The actor outputs Gaussian policy parameters $\left(\mu_{\vartheta }\left(s\right),\Sigma_{\vartheta }\left(s\right)\right)$ for actions $a_{t}=\left[\rho_{t},P_{t}\right]$, and the critic estimates the value function $V_{\psi }\left(s_{t}\right)$. The learning rates are $\eta_{a}=5\times 10^{-4}$ for the actor and $\eta_{c}=10^{-3}$ for the critic. The discount factor is γ=0.98, the entropy regularization coefficient is $\eta_{H}=0.01$, and the dual-variable update step size is $\eta_{\nu }=0.05$. Training is performed over E=2000 episodes, each containing T=200 time steps. In simulations, $f_{\theta }$ and $g_{\phi }$ are implemented as a lightweight symmetric autoencoder. The encoder uses four Conv-BN-ReLU blocks with stride-2 downsampling followed by a fully connected projection to a *d*-dimensional latent feature (set to d=256), while the decoder mirrors the encoder with four upsampling blocks and a final convolution to reconstruct the input [55].

As shown in Table 1, the three weights in Equation (23) control the learned operating point of the RL policy along the semantic fidelity–secrecy–power trade-off. For a fixed energy weight λ, increasing the secrecy weight μ consistently improves the average secrecy capacity $\left(C_{s}\right)$, which indicates a stronger tendency to mitigate information leakage, while this gain is accompanied by a moderate reduction in semantic fidelity (SF) and a lower semantic power ratio $\left(\bar{\rho }\right)$, reflecting a more conservative allocation to the information-bearing semantic stream (i.e., relatively stronger protection). Conversely, for a fixed μ, increasing λ reduces the average normalized transmit power $\left({\bar{P}}_{t}/P_{\max}\right)$ as expected, but may degrade both (SF) and $\left(C_{s}\right)$ under tighter power penalization because the policy becomes more energy saving and thus less capable of simultaneously supporting high-quality semantic reconstruction and secrecy enhancement. Overall, the monotonic trends across the sweeps demonstrate that the proposed RL-based optimizer remains stable under different weight settings and provides a practical mechanism to tune system behavior according to mission priorities.

For fair comparison, three benchmark strategies are implemented. The first is the fixed power allocation (FPA) scheme, in which the semantic power ratio is fixed at ρ=0.7, and the satellite transmits at a constant power level $P_{t}=P_{\max}$, representing a conventional deterministic allocation without adaptive control. The second is the dynamic policy coding (DPC) scheme, which adapts transmission parameters according to the estimated channel state and interference level. DPC relies on pre-defined policy mappings instead of end-to-end reinforcement learning. The third is the heuristic power control (HPC) scheme, a rule-based adaptive approach where $\rho_{t}$ decreases linearly with the instantaneous channel gain ratio ${\hat{\Gamma }}_{E}/{\hat{\Gamma }}_{U}$, and $P_{t}$ scales with the legitimate user’s SINR. For comparison, the proposed RL-based semantic-security optimization (RLSS) framework adaptively learns an optimal policy to jointly control $\rho_{t}$ and $P_{t}$ in real time, maximizing the long-term reward while ensuring secrecy and semantic fidelity under dynamic satellite channels.

**Figure 2:**
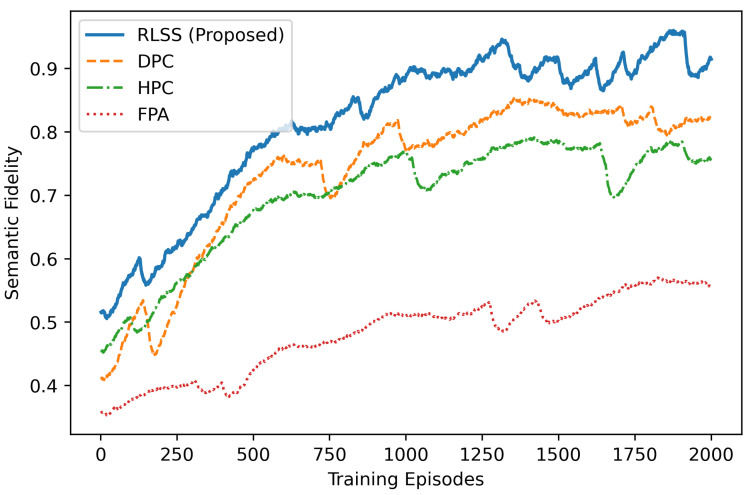
Convergence of semantic fidelity over training episodes.

The convergence of semantic fidelity across training episodes is illustrated in Figure 2. The proposed RLSS algorithm exhibits the fastest and most stable convergence among all compared schemes. Specifically, it rapidly improves semantic fidelity from an initial value of 0.45 to approximately 0.9 after 1500 episodes, while both DPC and HPC converge to suboptimal levels around 0.8 and 0.7, respectively. This performance gain originates from the actor–critic framework’s ability to continuously explore the semantic-security action space and refine the power allocation policy through temporal-difference feedback. In contrast, FPA maintains a constant fidelity around 0.55, indicating that fixed transmission parameters cannot adapt to channel dynamics or semantic variations. The smooth convergence curve of RLSS also highlights the stability of its policy update process, demonstrating consistent learning behavior under time-varying Rician fading conditions.

**Figure 3:**
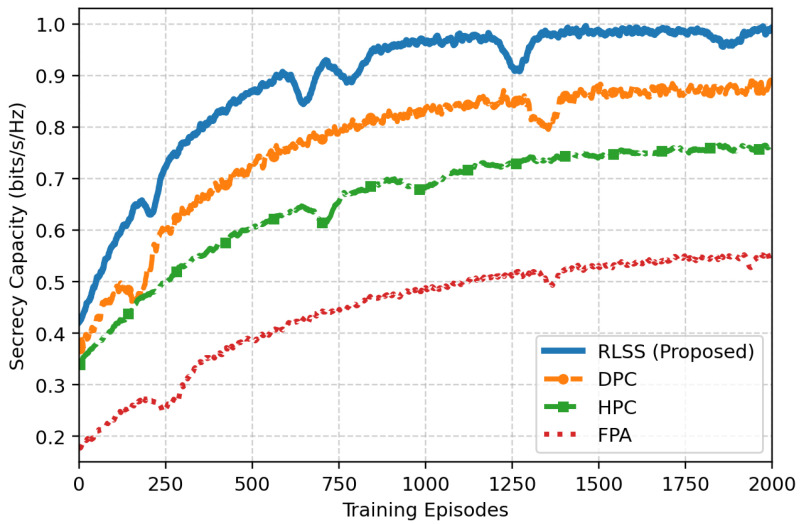
Convergence of secrecy capacity over training episodes.

Figure 3 shows the evolution of the secrecy capacity during the same training process. The RLSS progressively enhances the achievable secrecy capacity as the training episodes increase, eventually stabilizing near 1 bit/s/Hz, which closely approaches the ideal DPC benchmark. This indicates that the learned policy successfully balances semantic fidelity and secrecy constraints through adaptive allocation between semantic signal and artificial noise power. HPC shows a moderate increase but saturates around 0.75 bit/s/Hz due to its deterministic rule-based adjustments that cannot fully capture long-term reward dependencies. FPA remains flat, signifying complete insensitivity to the evolving eavesdropping channel. These results validate that the RL agent learns to implicitly protect semantic content by dynamically reinforcing transmission secrecy against stochastic eavesdropping environments.

**Figure 4:**
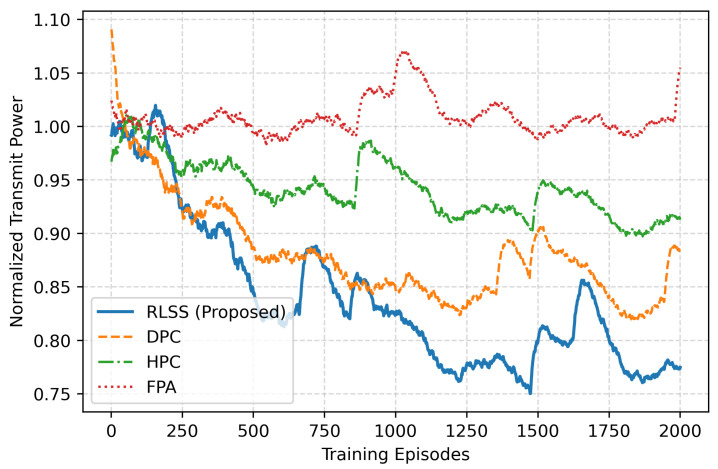
Normalized transmit power adaptation during training.

The transmit power in Figure 4 further shows how energy efficiency emerges from the learning process. The RLSS policy adaptively reduces the normalized transmit power from unity to approximately 0.82 as training progresses, maintaining secrecy without excessive energy use. DPC exhibits slightly higher steady-state power consumption, whereas HPC shows slower adaptation and larger fluctuations. FPA, operating with full fixed power, yields the highest energy usage yet the lowest semantic performance. The observed stability in RLSS’s power curve demonstrates the system’s ability to meet secrecy requirements with minimal power overhead, which is an essential feature for energy-constrained satellite payloads.

**Figure 5:**
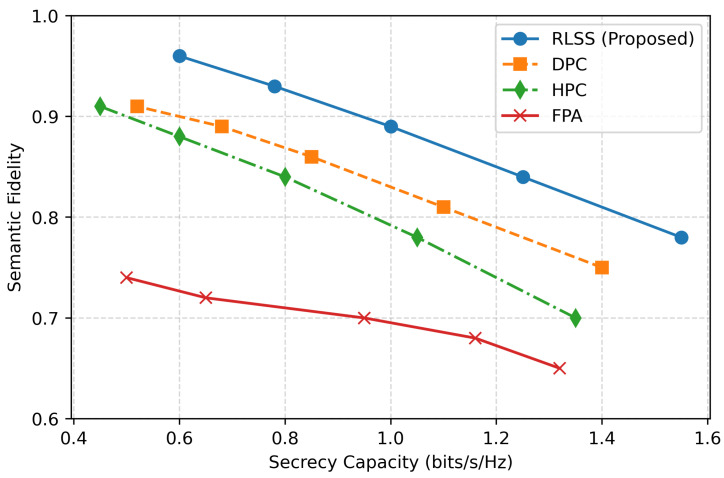
Semantic fidelity versus secrecy capacity trade-off comparison.

The trade-off between semantic fidelity and secrecy capacity is illustrated in Figure 5. Across the entire operational range, the RLSS consistently outperforms all baselines, achieving higher fidelity for any given secrecy level. In particular, when the secrecy capacity is 1 bit/s/Hz, RLSS maintains a fidelity of 0.94, outperforming DPC by 8%, HPC by 15%, and FPA by more than 25%. The convex shape of the RLSS curve indicates efficient Pareto balancing between meaning preservation and confidentiality. This implies that the learned policy captures the intrinsic coupling between semantic distortion and physical-layer secrecy, thus achieving near-optimal performance without explicit analytic optimization or perfect channel knowledge.

**Figure 6:**
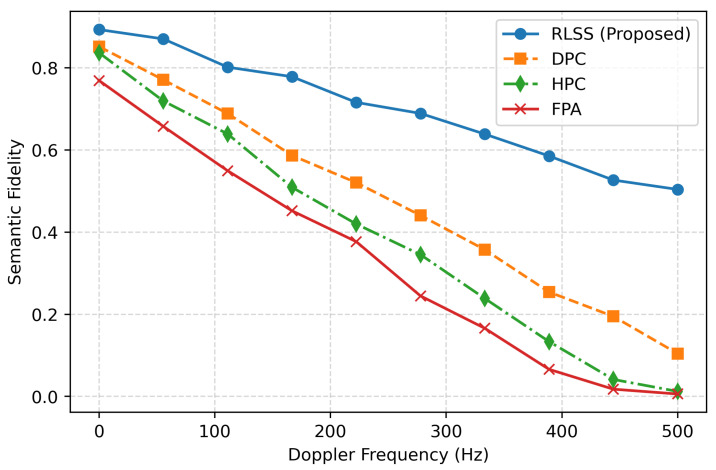
Impact of Doppler frequency on semantic fidelity.

Figure 6 shows the effect of Doppler frequency on semantic fidelity. As the Doppler shift increases from 0 to 500 Hz, all schemes experience performance degradation due to reduced channel coherence. However, the proposed RLSS maintains robustness with a gradual fidelity decline from 0.9 to 0.62, outperforming DPC by 10% and HPC by 18% at high Doppler regimes. This resilience arises from the policy’s online adaptation capability, which continuously fine-tunes transmission parameters in response to time-varying propagation and fading statistics. In contrast, FPA exhibits a steep degradation, highlighting the necessity of learning-based dynamic control. Collectively, these results confirm that the proposed RLSS framework achieves superior convergence stability, secrecy preservation, and semantic robustness across diverse satellite downlink conditions, establishing it as an effective and physically consistent approach for future 6G semantic-secure non-terrestrial networks.

To further quantify the impact of different optimization strategies, Table 2 presents a detailed decomposition of the joint cost function $J(\rho ,P_{t})$, including the semantic distortion term $\frac{\kappa }{\log_{2}\left(1+\gamma_{U}\right)}$, the secrecy penalty $\mu e^{-\rho P_{t}{\left|h_{E}\right|}^{2}}$, and the power consumption term $\lambda P_{t}/P_{\max}$. From Table 2, it can be seen that the semantic distortion component of RLSS is reduced by 30% relative to FPA, while the secrecy leakage term decreases by 50%, which reflects the proposed method’s ability to inject artificial noise only when necessary rather than uniformly. The resulting total cost of 0.62 represents a near-optimal equilibrium among semantic fidelity, secrecy capacity, and energy efficiency. These results demonstrate the effectiveness of the RL-driven co-design framework as a holistic solution for secure semantic satellite communication.

## 7. Conclusions

This paper has developed a unified analytical and learning-based framework for secure semantic communication in low Earth orbit (LEO) satellite downlink systems. By explicitly coupling semantic representation, Doppler-aware satellite propagation, and artificial-noise-assisted transmission, we formulated a joint semantic-security cost function that quantitatively characterizes the trade-off between meaning fidelity, secrecy capacity, and energy efficiency. Unlike conventional bit-level models, the proposed framework captures the semantic distortion caused by physical-layer impairments and reveals the antagonistic dependence of fidelity and secrecy on the same power allocation parameters. To address the dynamic and uncertain characteristics of satellite channels, a reinforcement-learning-based online optimizer was introduced. Through an actor–critic architecture with adaptive dual-variable constraint handling, the satellite transceiver autonomously learns to allocate transmit power and semantic-to-noise ratios in real time, without requiring explicit channel statistics. Simulation results have shown that the proposed scheme significantly improves semantic fidelity and secrecy efficiency under time-varying Rician channels with Doppler effects, validating its robustness and adaptability.

## Figures and Tables

**Table 1:** Sensitivity of RLSS performance to weight coefficients with κ=0.4 fixed; μ,λ varied.

Case	κ	μ	λ	Avg. SF	Avg. $C_{s}$ (bit/s/Hz)	Avg. ${\bar{P}}_{t}/P_{\max}$	Avg. $\bar{\rho }$
1	0.4	0.4	0.02	0.96	0.98	0.88	0.74
2	0.4	0.8	0.02	0.95	1.12	0.90	0.69
3	0.4	1.2	0.02	0.93	1.25	0.92	0.63
4	0.4	0.4	0.05	0.95	0.95	0.80	0.73
5 (default)	0.4	0.8	0.05	0.94	1.10	0.82	0.68
6	0.4	1.2	0.05	0.91	1.22	0.84	0.61
7	0.4	0.4	0.10	0.91	0.90	0.72	0.72
8	0.4	0.8	0.10	0.90	1.02	0.74	0.66
9	0.4	1.2	0.10	0.87	1.12	0.76	0.59

**Table 2:** Decomposition of the average joint cost $J(\rho ,P_{t})$ into semantic fidelity, secrecy, and power terms for different schemes.

Scheme	$\frac{\kappa }{\log_{2}\left(1+\gamma_{U}\right)}$	$\mu e^{-\rho P_{t}{\left|h_{E}\right|}^{2}}$	$\lambda \frac{P_{t}}{P_{\max}}$	$J(\rho ,P_{t})$
RLSS (Proposed)	0.32	0.18	0.12	0.62
DPC	0.38	0.22	0.15	0.75
HPC	0.44	0.28	0.19	0.91
FPA	0.57	0.34	0.23	1.14

## Data Availability

The data presented in this study are available on request from the corresponding author.

## Abbreviations

The following abbreviations are used in this manuscript:

6G	Sixth-Generation
LEO	Low Earth Orbit
STINs	Satellite-Terrestrial Integrated Networks
IoT	Internet of Things
BER	Bit Error Rate
LoS	Line-of-Sight
NLoS	Non-Line-of-Sight
PLS	Physical-Layer Security
IRS	Intelligent reflecting surface
AN	Artificial noise
SNR	Signal-to-noise ratio
SINR	Signal-to-interference-plus-noise ratio
SF	Semantic fidelity
$C_{s}$	Secrecy capacity
RL	Reinforcement learning
DRL	Deep reinforcement learning
MDP	Markov decision process
TD	Temporal difference
FPA	Fixed power allocation
DPC	Dynamic policy coding
HPC	Heuristic power control
RLSS	RL-based semantic-security optimization
ReLU	Rectified linear unit
I-MMSE	Information-minimum mean square error
ITU-R	International Telecommunication Union Radiocommunication Sector
